# Role of hypoxia-inducible factor-1α, carbonic anhydrase-IX, glucose transporter-1 and vascular endothelial growth factor associated with lymph node metastasis and recurrence in patients with locally advanced cervical cancer

**DOI:** 10.3892/ol.2015.3524

**Published:** 2015-07-23

**Authors:** KEITA IWASAKI, HIROMITSU YABUSHITA, TAIKI UENO, AKIHIKO WAKATSUKI

**Affiliations:** Department of Obstetrics and Gynecology, School of Medicine, Aichi Medical University, Nagakute, Aichi 480-1195, Japan

**Keywords:** hypoxia-inducible factor-1α, carbonic anhydrase-IX, glucose transporter-1, vascular endothelial growth factor, immunohistochemistry, cervical cancer

## Abstract

The aim of the present study was to determine whether the expression of hypoxia-inducible factor-1α (HIF-1α), carbonic anhydrase-IX (CA-IX), glucose transporter-1 (GLUT-1) or vascular endothelial growth factor (VEGF) was associated with the clinicopathological characteristics, lymph node metastasis or progression-free survival of patients with cervical cancer. Tumor tissue samples were obtained from 54 cervical cancer patients who had undergone radical hysterectomy. The expression of HIF-1α, CA-IX, GLUT-1 and VEGF was analyzed by immunohistochemical staining. Of the 54 cases, 28 were positive for HIF-1α, 35 for CA-IX, 40 for GLUT-1 and 23 for VEGF. It was revealed that HIF-1α expression was correlated with tumor stage and histology, CA-IX expression with tumor stage, tumor size, lymph node metastasis and lymph-vascular space involvement, GLUT-1 expression with tumor stage and lymph-vascular space involvement, and VEGF expression with microvessel density. The multivariate regression analysis indicated that CA-IX expression and lymph-vascular space involvement were independent variables associated with lymph node metastasis. Progression-free survival was shorter for patients who were positive for CA-IX or VEGF expression than for those who were negative for CA-IX or VEGF expression. The progression-free survival of patients treated with radiotherapy or chemo-radiotherapy following radical hysterectomy was also shorter for patients with positive CA-IX expression. These findings suggest that CA-IX expression is a possible risk factor for lymph node metastasis and disease recurrence in locally advanced cervical cancer patients.

## Introduction

The number of patients with cervical cancer has decreased as a result of cytological screening and DNA testing for the high-risk human papilloma virus. However, cervical cancer remains a considerable burden, with 500,000 new cases and 250,000 mortalities each year worldwide (1). The important prognostic factors for cervical carcinoma are represented by the International Federation of Gynecology and Obstetrics (FIGO) stage (2), lymph node metastasis and the pathological features of the primary tumor, including tumor size, depth of stromal invasion, histological type and lymph-vascular space involvement. Of these factors, lymph node metastasis has demonstrated the most marked association with disease recurrence in early-stage cases (3–5). An increasing requirement exists to identify biomarkers that may be able to predict treatment responses and patient survival. In addition, biological variables and gene profiles associated with aggressive clinical behavior may aid in establishing optimal therapeutic strategies for early-stage cervical cancers that present with high-risk factors.

Hypoxia is an important process in tumor biology, as it induces an aggressive phenotype with increased invasiveness, leads to the formation of metastases and results in poorer patient survival (6,7). In addition, hypoxic malignant cells exhibit increased resistance to chemotherapy and radiotherapy (8,9). Cells react to hypoxic conditions by altering their metabolism and activating specific survival genes. Hypoxia inducible factor-1 (HIF-1) has an important role in the adaptive cellular response to hypoxia (10). HIF-1 is a transcription factor composed of the basic helix-loop-helix DNA-binding proteins, HIF-1α and HIF-1β. Under normoxia, HIF-1α is hydroxylated by prolyl hydroxylases. Hydroxylated HIF-1α is then recognized by the von Hippel Lindau protein, ubiquitinated and targeted to the proteasome for degradation. However, during hypoxia, this process is inhibited (11). Instead, following nuclear translocation, the stabilized HIF-1α heterodimerizes with HIF-1β to transactivate target genes (12). Furthermore, glycolytic enzymes, glucose transporters, growth factors and genes that are involved in gluconeogenesis are activated under hypoxia. These molecules enable the cell to survive hypoxic stress by increasing oxygen delivery through angiogenesis and by inducing a switch to anaerobic glycolysis (10,12–14). HIF-1α expressed under hypoxic conditions has a significant role in these processes, as it activates the expression of target genes, such as carbonic anhydrase-IX (CA-IX), which has a role in pH regulation (15), glucose transporter-1 (GLUT-1), which is a transmembrane glucose transporter (16), and vascular endothelial growth factor (VEGF), which is involved in angiogenesis (17).

CA-IX is a transmembrane glycoprotein that catalyzes the reversible hydration of carbonic dioxide to carbonic acid. CA-IX is therefore important for pH regulation and the elimination of the hypoxia-generated acid load during glycolysis. Previous data has established that CA-IX is overexpressed in a number of human cancers (18). Further studies have demonstrated that elevated levels of CA-IX are predictive of hypoxia in a variety of cancers and are associated with a poorer prognosis (19,20).

The membrane-bound glycoprotein, GLUT-1, is a high-affinity glucose transporter responsible for the regulation of glucose uptake (21). The expression of GLUT-1 is upregulated during hypoxia and other conditions that induce an increased dependence on the use of anaerobic glycolysis as an energy source (22). GLUT-1 is undetectable in the majority of normal epithelial tissues and benign epithelial tumors, but is expressed at a significantly higher level in a range of human carcinomas (21).

VEGF is a highly-specific mitogen for vascular endothelial cells. In response to hypoxia, the expression of VEGF is upregulated by activated oncogenes and a number of cytokines. VEGF initiates endothelial cell proliferation and angiogenesis, as well as the permeabilization of tumor blood vessel (23).

The majority of locally advanced cervical cancers can be cured with radical surgery and chemo-radiotherapy. However, patients with persistent or recurrent disease have limited treatment options, and novel therapeutic strategies, including immunotherapy, are required for such patients (24).

It has been reported that vaccination with CA-IX-derived peptides is an effective immunotherapy for renal cell carcinoma patients (25), and that the addition of bevacizumab, a humanized anti-VEGF monoclonal antibody, to conventional therapy for patients with cervical cancer improves survival rates (26).

In anticipation of the development of novel therapeutic strategies for locally advanced cervical cancer, the present study aimed to determine whether HIF-1α, CA-IX, GLUT-1 or VEGF were associated with the clinicopathological characteristics, lymph node metastasis or progression-free survival of patients with cervical carcinoma.

## Materials and methods

### 

#### Clinical samples

Formalin-fixed, paraffin-embedded tumor tissues were obtained from 54 patients with locally advanced cervical carcinoma. All patients had attended the Gynecology Clinic at the Aichi Medical University Hospital (Nagakute, Japan), were diagnosed with cervical carcinoma and had undergone a radical hysterectomy. The mean age of the patients was 49.4±11.9 years old (range, 25 to 72 years old). The clinicopathological characteristics of the patients and the adjuvant therapies used following radical surgery are shown in Table I. The present study was approved by the regional ethics committee of Aichi Medical University, School of Medicine (Nagakute, Japan). Written informed consent was obtained from all participants prior to study enrollment.

#### Immunohistochemistry

The 3-µm thick tumor sections were first deparaffinized and rehydrated. Subsequent to microwave processing in 10 mM citrate buffer (pH 6.0) for 25 min, the sections were incubated for 30 min in methanol containing 0.5% H_2_O_2_. Following incubation in normal goat serum for 1 h at room temperature to block non-specific binding, the slides were incubated at 4°C overnight with the following primary antibodies: Mouse anti-HIF-1α antiserum (dilution, 1:100; product no. ab1; Abcam, Tokyo, Japan), rabbit anti-CA-IX antiserum (dilution, 1:1000; product no. ab15086; Abcam), rabbit anti-VEGF antiserum (dilution, 1:200; product no. ab46154; Abcam) and rabbit anti-GLUT-1 antiserum (dilution, 1:200; product no. ab14683; Abcam). Following incubation with the primary antibody, the Envision Polymer Component (ChemMate ENVISION kit; Dako, Kyoto, Japan) was added to the slides for 30 min at room temperature. The horseradish peroxidase reaction was then developed using 3,3′-diaminobenzidine tetrahydrochloride (Katayama Chemical Industries Co., Ltd., Osaka, Japan). Finally, for the microscopic examination, sections were counterstained with hematoxylin (magnification, ×200; Olympus BX43; Olympus Corporation, Tokyo, Japan). The tissues were defined as having positive expression when >50% of the tumor cells demonstrated intense staining.

#### Examination of microvessel density and lymph-vascular space involvement

Blood vessels were identified by immunohistochemical staining of the endothelial cells using mouse anti-cluster of differentiation (CD)34 antiserum (dilution, 1:25; product no. ICO 115; Cell Signaling Technology Japan, Tokyo, Japan), according to the aforementioned procedure. The total number of vessels in each case was taken to be the total sum of vessels counted in each of 10 microscopic fields. The vessels were analyzed at ×400 magnification. The microvessel density was defined as the average number of microvessels per field, calculated from the total number of microvessels in 10 fields.

For the assessment of lymph-vascular space involvement, lymph and blood vessels were immunohistochemically stained with the already diluted mouse monoclonal antibody against D2-40 (dilution, 1:25; product no. 413451; Nichirei Biosciences Inc., Tokyo, Japan) and the mouse anti-CD34 antiserum (dilution, 1:25; Cell Signaling Technology Japan) according to the aforementioned procedure. The presence of carcinoma cells in the lymph and blood vessels indicated positive lymph-vascular space involvement.

#### Statistical analysis

Stat View-J version 5 (Apple Inc., Cupertino, CA, USA) was used for the statistical analyses. The statistical significance of the differences between categories of expression was analyzed using Fisher's exact test. The potential significance of plural risk factors for lymph node metastasis was analyzed using a logistic regression test. Progression-free survival was analyzed by the Kaplan-Meier method and a log-rank test. P<0.05 was used to indicate a statistically significant difference.

## Results

**Figure 1.**
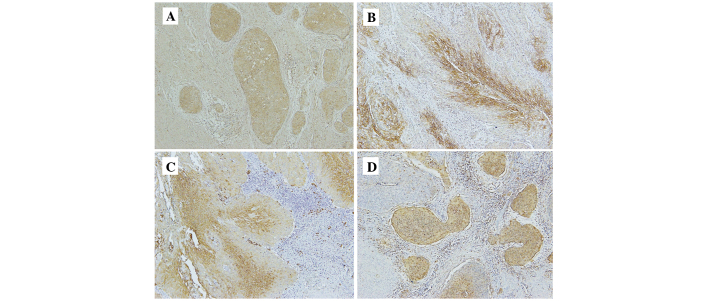
Representative images of the immunohistochemical visualization of (A) hypoxia inducible factor-1α, (B) carbonic anhydrase-IX, (C) glucose transporter-1 and (D) vascular endothelial growth factor in cervical squamous cell carcinoma. Fixed cells were stained with specific antibodies and horseradish-peroxidase secondary antibodies, and then counterstained with hematoxylin (magnification, ×200).

The expression of HIF-1α, CA-IX, GLUT-1 and VEGF in cervical squamous cell carcinomas was analyzed immunohistochemically. HIF-1α was observed in the cell nuclei and cytoplasm of the tumor cells, whereas CA-IX, GLUT-1 and VEGF were predominantly localized in the cell membrane and cytoplasm. HIF-1α and VEGF stained homogenously throughout the cancer nest, whereas CA-IX and GLUT-1 were localized in the center (Fig. 1).

Of the 54 cases, 28 were positive for HIF-1α expression, 35 for CA-IX, 40 for GLUT-1 and 23 for VEGF. Analysis of the correlation between the expression of these different factors indicated that HIF-1α was significantly associated with CA-IX, but not with GLUT-1 or VEGF. In addition, CA-IX expression was correlated with GLUT-1 and VEGF, but no association was identified between GLUT-1 and VEGF (Table II).

The expression of these factors was then correlated with the tumor parameters. A higher expression level of HIF-1α, CA-IX and GLUT-1 was observed in stage II cases compared with stage I cases. By contrast, VEGF was not associated with the tumor stage. HIF-1α was the only factor to demonstrate a higher expression level in adenocarcinomas compared with the squamous cell carcinomas. None of the other factors exhibited an association with expression levels and tumor histology. CA-IX was the only factor to demonstrate an association with tumor size. CA-IX was more highly expressed in tumors measuring ≥4 cm compared with those measuring <4 cm. Furthermore, CA-IX was also the only factor to be correlated with lymph node metastasis, being more highly expressed in these cases. Only CA-IX and GLUT-1 exhibited an association with lymph-vascular space involvement, and only VEGF was correlated with microvessel density. A higher expression level of VEGF was observed in tumors with high microvessel density than in those with low microvessel density (Table III).

The association between the co-expression levels of these factors and the tumor parameters was then investigated. Higher co-expression of HIF-1α and CA-IX was observed in stage II cases compared with stage I cases. In addition, higher co-expression of CA-IX and GLUT-1 was observed in stage II cases compared with stage I cases, as well as in tumors ≥4 cm, in cases with lymph node metastasis and in tumors with lymph-vascular space involvement. Finally, a higher co-expression level of CA-IX and VEGF was observed in tumors ≥4 cm and in tumors with a higher microvessel density (Table IV).

The multivariate regression analysis revealed that CA-IX expression and lymph-vascular space involvement were independent variables associated with lymph node metastasis in patients with cervical cancer (Table V).

**Figure 2.**
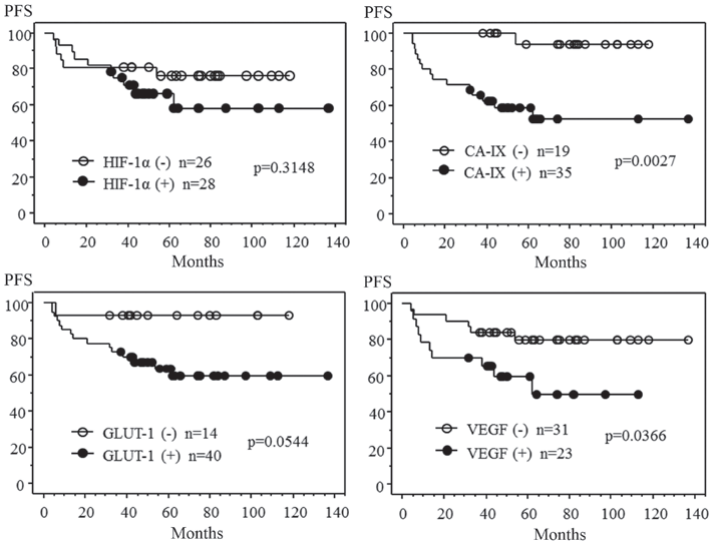
Correlation of PFS with HIF-1α, CA-IX, GLUT-1 and VEGF expression levels. Expression of HIF-1α, CA-IX, GLUT-1 and VEGF was immunohistochemically analyzed in 54 patients with cervical cancer. PFS time over 135 months was analyzed using the Kaplan-Meier method and a log-rank test. HIF-1α, hypoxia-inducible factor-1α; CA-IX, carbonic anhydrase-IX; GLUT-1, glucose transporter-1; VEGF, vascular endothelial growth factor; PFS, progression-free survival.

**Figure 3.**
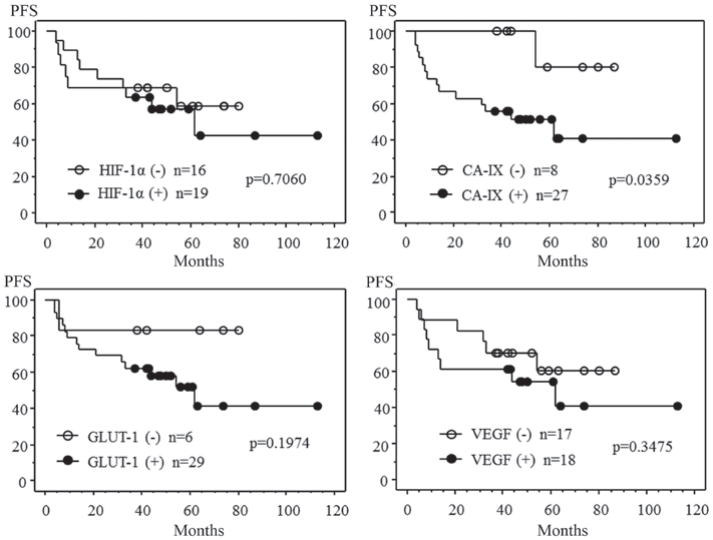
Correlation of progression-free survival with HIF-1α, CA-IX, GLUT-1 and VEGF expression levels in cervical cancer patients treated with radiotherapy or chemo-radiotherapy following radical hysterectomy. Expression of HIF-1α, CA-IX, GLUT-1 and VEGF was immunohistochemically analyzed in 35 patients with cervical cancer. Progression-free survival time over 110 months was analyzed using the Kaplan-Meier method and a log-rank test. HIF-1α, hypoxia-inducible factor-1α; CA-IX, carbonic anhydrase-IX; GLUT-1, glucose transporter-1 glucose transporter-1; VEGF, vascular endothelial growth factor; PFS, progression-free survival.

**Figure 4.**
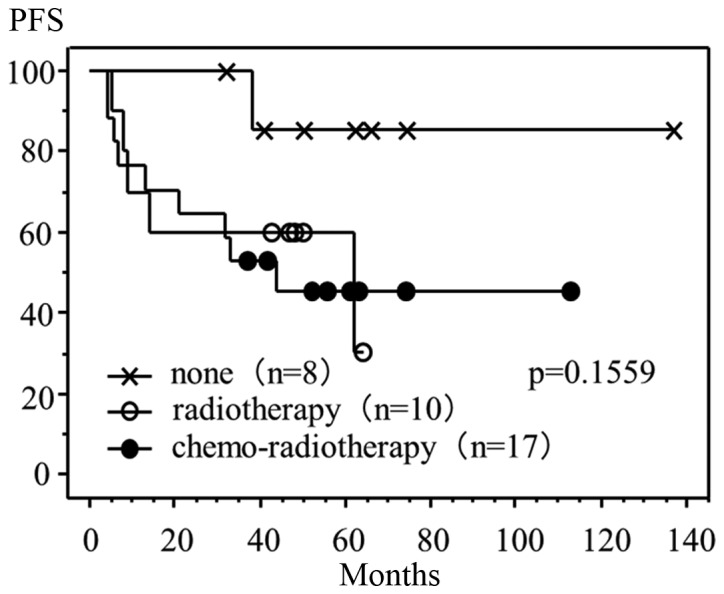
Correlation of positive CA-IX expression with progression-free survival in patients who received adjuvant therapies following radical hysterectomy. The progression-free survival time of 35 patients with cervical cancer, who tested positive for CA-IX expression and who received the indicated adjuvant therapy after radical hysterectomy, was analyzed using the Kaplan-Meier method and a log-rank test. CA-IX, carbonic anhydrase-IX; PFS, progression-free survival.

The Kaplan-Meier analyses indicated that progression-free survival time was shorter in patients with a larger tumor size, positive lymph node metastasis, positive lymph-vascular space involvement and a higher tumor microvessel density (data not shown). Progression-free survival time was also shorter in patients positive for CA-IX or VEGF expression than in those negative for CA-IX or VEGF expression. However, progression-free survival time demonstrated no association with the expression of HIF-1α or GLUT-1 (Fig. 2). In the 35 patients treated with radiotherapy or chemo-radiotherapy following radical hysterectomy, progression-free survival time was also shorter for those individuals positive for CA-IX expression compared with those negative for CA-IX expression (Fig. 3), and for patients with positive lymph-vascular space involvement compared with those negative for lymph-vascular space involvement (data not shown). However, progression-free survival time exhibited no correlation with tumor size, lymph node metastasis, microvessel density (data not shown) or VEGF expression (Fig. 3). Finally, of the 35 cases positive for CA-IX expression, progression-free survival time demonstrated no association with the performance of adjuvant therapies following radical hysterectomy (Fig. 4).

## Discussion

Tumor hypoxia is a factor known to be associated with genetic instability, resistance to apoptosis, invasive growth and metastatic spread. In the present study, the expression of HIF-1α, CA-IX, GLUT-1 and VEGF was examined in order to determine whether these molecules may be useful tissue biomarkers of tumor hypoxia. The expression of these potential markers was examined immunohistochemically in biopsies obtained from patients with locally advanced cervical carcinoma who had undergone radical hysterectomy followed by post-surgical radiotherapy or chemo-radiotherapy. The most important findings of this study were that the expression of CA-IX and the presence of lymph-vascular space involvement were associated with lymph node metastasis, and that the expression of CA-IX was clearly associated with disease recurrence, regardless of the treatment modality.

A previous retrospective study that analyzed 130 squamous cell cervical carcinoma biopsies revealed that the expression of CA-IX was an independent prognostic indicator of poor overall survival and metastasis-free survival following definitive radiotherapy (20). In a further study by the Gynecological Oncology Group, the expression of CA-IX was immunohistochemically analyzed in tumor biopsies obtained from 166 women who had undergone a radical hysterectomy for stage Ia2-IIa cervical cancer that had presented with pathological findings of lymph node metastases, parametrial involvement or positive surgical margins. The patients in the study received either adjuvant pelvic radiotherapy alone, or adjuvant pelvic radiotherapy combined with concomitant cisplatin- and 5-fluorouracil-based chemotherapy (27,28). A high expression of CA-IX has been identified to be significantly associated with tumor size and depth of stromal invasion in patients with cervical cancer, and is also an independent predictor of poor survival (28). In addition, it has been demonstrated that the absence of GLUT-1 immunostaining is associated with improved metastasis-free survival in patients who receive definitive radiotherapy (29). VEGF immunostaining has also been reported to be significantly correlated with disease-free survival and overall survival in patients treated with neoadjuvant chemotherapy or primary radiotherapy (30,31). Of the VEGF isoforms, it has been established that VEGF-C is essential for lymphangiogenesis and the lymphatic spread of tumors (32). A previous study revealed that VEGF-C expression is higher in patients positive for lymph node metastasis than in those negative for lymph node metastasis. Furthermore, the results indicated that this variable was an independent predictor of lymph node status, and that the univariate, but not the multivariate analysis of patients whose tumors were positive for VEGF-C mRNA revealed a shorter disease-free survival time (33).

The present study revealed that CA-IX was associated with the expression of HIF-1α, GLUT-1 and VEGF. However, no correlation was identified between the expression of HIF-1α and GLUT-1, between HIF-1α and VEGF, or between GLUT-1 and VEGF. The results also demonstrated an association between these markers and specific tumor parameters. HIF-1α expression was associated with the FIGO stage and histological type, but not with tumor size, lymph node metastasis, lymph-vascular involvement or microvessel density. CA-IX expression was associated with the FIGO stage, tumor size, lymph node metastasis and lymph-vascular space involvement. GLUT-1 expression was associated with the FIGO stage and lymph-vascular involvement, and VEGF expression was associated with microvessel density. The co-expression of CA-IX and GLUT-1 was associated with the FIGO stage, tumor size, lymph node metastasis and lymph-vascular space involvement, and the co-expression of CA-IX and VEGF was associated with tumor size and microvessel density.

These findings suggest that lymph node metastasis following lymph-vascular space involvement may be associated with pH regulation and anaerobic glycolysis, which under the hypoxic conditions of the tumor, is assisted by CA-IX and GLUT-1. In addition, the results indicate that lymph node metastasis is possibly associated with VEGF-induced angiogenesis.

The multivariate regression analysis revealed that CA-IX expression and lymph-vascular space involvement were independent variables associated with lymph node metastasis in patients with cervical cancer. These findings suggest: i) That the pH regulation induced by CA-IX under hypoxic conditions may be associated with lymph node metastasis; ii) that CA-IX can function as a biomarker with the ability to predict lymph node metastasis; and iii) that CA-IX is potential molecular target for the treatment of cervical cancer.

The Kaplan-Meier analyses indicated that the progression-free survival time was shorter for patients with positive CA-IX or VEGF expression than for those with negative CA-IX or VEGF expression. A similar correlation between CA-IX expression and progression-free survival time was identified in patients treated with radiotherapy or chemo-radiotherapy following radical surgery. However, no association was established between progression-free survival time and the performance of adjuvant therapies in patients positive for CA-IX expression following radical surgery.

These results suggest that CA-IX expression, as well as lymph node metastasis, larger tumor size and lymph-vascular space involvement, are important predictive factors associated with disease recurrence in locally advanced cervical cancer. Therefore, it is hoped that novel therapeutic approaches that target CA-IX can be developed, as at present, no improvement in progression-free survival time in cases positive for CA-IX expression has been observed, even when adjuvant radiotherapy or chemo-radiotherapy is administered following radical surgery.

Although extremely few targeted therapies have been evaluated for cervical carcinoma, the findings of the present study indicate a possibility for molecular therapies targeted at CA-IX or VEGF. It was previously reported that vaccination with CA-IX-derived peptides was an effective immunotherapy for renal cell carcinoma patients (25). The results of the present study suggest that vaccination with CA-IX-derived peptides may also present a novel form of therapy for cervical cancer patients. Additionally, it has been reported that the addition of bevacizumab, a humanized anti-VEGF monoclonal antibody, to the conventional therapy of patients with cervical cancer improves survival (26). The results of the present study demonstrate that VEGF expression in cervical cancer is an important risk factor associated with disease recurrence. Therefore, anti-VEGF immunotherapy may also be a useful therapeutic approach for the treatment of patients with cervical cancer.

In conclusion, the findings of the present study indicate that CA-IX is a possible risk factor for lymph node metastasis and disease recurrence in locally advanced cervical cancer patients. The pH regulation induced by CA-IX expression under hypoxic conditions may be associated with lymph node metastasis and a poor progression-free survival time. Therefore, it is hypothesized that the vaccination of cervical carcinoma patients whose tumors express CA-IX with CA-IX-derived peptides may prove to be an effective therapy.

## Figures and Tables

**Table I.** Characteristics of the 54 cervical cancer patients who underwent radical hysterectomy followed by adjuvant radiotherapy or chemo-radiotherapy.

		Adjuvant therapy following radical hysterectomy (n)		
		
Characteristic	n	None	Radiotherapy	Chemo-radiotherapy
FIGO stage				
Ib1	24	18	4	2
Ib2	15	1	5	9
IIa1	1	0	0	1
IIa2	5	0	2	3
IIb	9	0	2	7
Histology				
Squamous cell carcinoma	34	9	9	16
Adenocarcinoma	20	10	4	6
Tumor size, cm				
<4	27	18	5	4
≥4	27	1	8	18
Lymph node metastasis				
Negative	36	18	9	9
Positive	18	1	4	13
Lymph-vascular space involvement				
Negative	38	19	8	11
Positive	16	0	5	11
Microvessel density, vessels/×400 field				
≤5	25	13	4	8
>5	29	6	9	14
Total	54	19	13	22

FIGO, International Federation of Gynecology and Obstetrics.

**Table II.** Co-expression of HIF-1α, CA-IX, GLUT-1 and VEGF.

A, HIF-1α			

Parameter	Negative (n=26)	Positive (n=28)	P-value
CA-IX			0.0096
Negative (n=19)	14	5	
Positive (n=35)	12	23	
GLUT-1			0.4399
Negative (n=14)	8	6	
Positive (n=40)	18	22	
VEGF			0.5541
Negative (n=31)	16	15	
Positive (n=23)	10	13	

B, CA-IX			

Parameter	Negative (n=19)	Positive (n=35)	P-value

GLUT-1			0.0081
Negative (n=14)	9	5	
Positive (n=40)	10	30	
VEGF			0.0033
Negative (n=31)	16	15	
Positive (n=23)	3	20	

C, GLUT-1			

Parameter	Negative (n=14)	Positive (n=40)	P-value

VEGF			0.347
Negative (n=31)	10	21	
Positive (n=23)	4	19	

HIF-1α, hypoxia-inducible factor-1α; CA-IX, carbonic anhydrase-IX; GLUT-1, glucose transporter-1; VEGF, vascular endothelial growth factor; ns, not significant.

**Table III.** Association of HIF-1α, CA-IX, GLUT-1 and VEGF expression levels with FIGO stage, histological type, tumor size, lymph node metastasis, lymph-vascular space involvement and microvessel density.

		Positive immunohistochemical expression [n, (%)]			
		
Characteristic	n	HIF-1α	CA-IX	GLUT-1	VEGF
FIGO stage					
I	39	16 (41%)	22 (56%)	25 (64%)	15 (38%)
II	15	12 (80%)	13 (87%)	15 (100%)	8 (53%)
P-value		0.0102	0.0370	0.0070	0.3222
Histology					
SCC	34	13 (38%)	20 (59%)	28 (82%)	13 (38%)
Adenocarcinoma	20	15 (75%)	15 (75%)	12 (60%)	10 (50%)
P-value		0.0090	0.2293	0.0703	0.3985
Tumor size, cm					
<4	27	13 (48%)	13 (48%)	17 (63%)	8 (30%)
≥4	27	15 (56%)	22 (81%)	23 (85%)	15 (56%)
P-value		0.5863	0.0103	0.0624	0.0525
Lymph-node metastasis					
Negative	36	18 (50%)	20 (56%)	24 (67%)	17 (47%)
Positive	18	10 (56%)	15 (83%)	16 (89%)	6 (33%)
P-value		0.7001	0.0439	0.0790	0.3306
Lymph-vascular space involvement					
Negative	38	18 (47%)	21 (55%)	25 (66%)	15 (39%)
Positive	16	10 (63%)	14 (88%)	15 (94%)	8 (50%)
P-value		0.3095	0.0235	0.0323	0.4750
Microvessel density, vessels/×400 field					
≤5	25	11 (44%)	13 (52%)	16 (64%)	6 (24%)
>5	29	17 (59%)	22 (76%)	24 (83%)	17 (59%)
P-value		0.2836	0.0671	0.1168	0.0103

HIF-1α, hypoxia-inducible factor-1α; CA-IX, carbonic anhydrase-IX; GLUT-1, glucose transporter-1; VEGF, vascular endothelial growth factor; FIGO, International Federation of Gynecology and Obstetrics; SCC, squamous cell carcinoma; ns, not significant.

**Table IV.** Association of HIF-1α, CA-IX, GLUT-1 and VEGF co-expression with the FIGO stage, histological type, size, lymph node metastasis, lymph-vascular space involvement and microvessel density of the tumors.

		Immunohistochemical co-expression [n, (%)]		
		
Characteristic	n	HIF-1α + CA-IX	CA-IX + GLUT-1	CA-IX + VEGF
FIGO stage				
I	39	13 (33%)	17 (44%)	12 (31%)
II	15	10 (67%)	13 (87%)	8 (53%)
P-value		0.0349	0.0056	0.2074
Histology				
SCC	34	11 (32%)	20 (59%)	11 (32%)
Adenocarcinoma	20	12 (60%)	10 (50%)	9 (45%)
P-value		0.0861	0.5798	0.3934
Tumor size, cm				
<4	27	10 (37%)	9 (33%)	6 (22%)
≥4	27	13 (48%)	21 (78%)	14 (52%)
P-value		0.5826	0.0023	0.0473
Lymph-node metastasis				
Negative	36	14 (39%)	16 (44%)	14 (39%)
Positive	18	9 (50%)	14 (78%)	6 (33%)
P-value		0.5615	0.0239	0.7712
Lymph-vascular space involvement				
Negative	38	14 (37%)	17 (45%)	12 (32%)
Positive	16	9 (56%)	13 (81%)	8 (50%)
P-value		0.2350	0.0176	0.2301
Microvessel density, vessels/×400 field				
≤5	25	9 (36%)	11 (44%)	4 (16%)
>5	29	14 (48%)	19 (66%)	16 (55%)
P-value		0.4170	0.1700	0.0044

HIF-1α, hypoxia-inducible factor-1α; CA-IX, carbonic anhydrase-IX; GLUT-1, glucose transporter-1; VEGF, vascular endothelial growth factor; FIGO, International Federation of Gynecology and Obstetrics; SCC, squamous cell carcinoma; ns, not significant.

**Table V.** Multivariate analyses of variables associated with lymph node metastasis in 54 patients with cervical cancer.

Variables	Odds ratio	95% CI	P-value
FIGO stage (II vs. I)	8.486	0.794–90.749	ns
Histology (adenocarcinoma vs. squamous cell carcinoma)	0.289	0.034–2.427	ns
Tumor size (≥4 cm vs. <4 cm)	0.16	0.012–2.181	ns
Lymph-vascular space involvement (positive vs. negative)	39.413	2.792–556.363	0.0065
Microvessel density (>5 vs. ≤5, vessels/×400 field)	6.531	0.648–65.781	ns
HIF-1α expression (positive vs. negative)	0.247	0.031–1.952	ns
CA-IX expression (positive vs. negative)	33.217	1.016–1085.947	0.0489
GLUT-1 expression (positive vs. negative)	0.421	0.019–9.189	ns
VEGF expression (positive vs. negative)	0.134	0.002–1.574	ns

HIF-1α, hypoxia-inducible factor-1α; CA-IX, carbonic anhydrase-IX; GLUT-1, glucose transporter-1; VEGF, vascular endothelial growth factor; FIGO, International Federation of Gynecology and Obstetrics; ns, not significant; CI, confidence interval.
